# NSC‐34 motor neuron‐like cells are sensitized to ferroptosis upon differentiation

**DOI:** 10.1002/2211-5463.12577

**Published:** 2019-02-23

**Authors:** Alejandra M. Martinez, Jovan Mirkovic, Zofia A. Stanisz, Fahmida S. Patwari, Wan Seok Yang

**Affiliations:** ^1^ Department of Biological Sciences St John's University Queens NY USA

**Keywords:** differentiation, ferroptosis, glutathione peroxidase 4, Gpx4, motor neuron, NSC‐34, transsulfuration pathway

## Abstract

Ferroptosis is a form of regulated cell death that is driven by lethal accumulation of lipid peroxides upon inhibition of glutathione peroxidase 4 (GPx4). Deletion of the *Gpx4* gene in mice revealed that neurons are sensitive to ferroptosis *in vivo*. However, few studies have been conducted on ferroptosis regulation in neurons. Here, we report that cells of a motor neuron‐like cell line, NSC‐34, became more sensitive to ferroptosis upon differentiation into a more motor neuron‐like condition. We identified three factors that influence ferroptosis sensitivity under differentiation conditions: low serum antioxidants, decreased GPx4 protein amount, and inhibition of the transsulfuration pathway. Our results support the hypothesis that neurons, especially motor neurons, are sensitive to ferroptosis, and suggest that ferroptosis in a neuronal context should be investigated further to develop strategies for neuroprotection.

**Abbreviations**
EEA: excitotoxic amino acid
Fer‐1: ferrostatin‐1
GPx4: glutathione peroxidase 4
HBSS: Hanks’ balanced salt solution
MEM: minimal essential medium
Nec‐1s: necrostatin‐1 stable
PARP: poly(ADP‐ribose) polymerase
PPG: propargylglycine
PUFA: polyunsaturated fatty acid
RA: retinoic acid
ROS: reactive oxygen species
xCT: system $x_{c}^{-}$
zVAD‐fmk: benzyloxycarbonyl‐phenylalanyl‐alanyl‐fluoromethyl ketone

Ferroptosis is a form of regulated cell death that is characterized by lethal accumulation of lipid peroxides in sensitive cells 1. It is genetically, biochemically, and morphologically distinctive from other cell death modalities and has been implicated in various pathological conditions and therapeutic strategies 2, 3.

Two classes of small molecule ferroptosis inducers have been identified. Class 1 ferroptosis inducers include erastin, glutamate, sorafenib, and sulfasalazine and inhibit system $x_{c}^{-}$ (xCT), a plasma membrane cystine–glutamate antiporter 4. Class 2 inducers include RSL3, ML162 and DPI compounds and directly inhibit glutathione peroxidase 4 (GPx4), an enzyme that removes lipid peroxides within cells 5. As inhibition of xCT leads to the depletion of cellular glutathione, an essential cofactor for GPx4 enzyme, both ferroptosis‐inducing mechanisms converge on inhibition of GPx4, which highlights the importance of GPx4 in ferroptosis regulation.

Genetic ablation of *GPx4* in mouse models resulted in embryonic lethality demonstrating the essentiality of GPx4 for survival 6, 7. Subsequent generation of conditional GPx4 knockout mice allowed further *in vivo* studies on the functional role of GPx4 and ferroptosis 8, 9, 10. It was reported that conditional whole‐body deletion of GPx4 in adult mice accompanied massive lipid peroxidation and cell death in various tissues – loss of neurons in brain was particularly notable 9. Neuron‐specific deletion of GPx4 in adult mice produced a severe neurodegenerative phenotype with rapid onset and progression of paralysis and death 8, 10. As the phenotype suggested, it turned out that motor neurons of the mice were particularly susceptible to ferroptosis by GPx4 deletion 10. The result implies that ferroptosis inhibition by GPx4 is essential for motor neuron survival.

We were interested in analyzing motor neuron susceptibility to ferroptosis because there has been no information about ferroptosis regulation in a motor neuron context. Degeneration of motor neurons is the main cause of motor neuron diseases such as amyotrophic lateral sclerosis; therefore, studies on the ferroptotic cell death pathway in motor neurons may lead to the development of therapeutic strategies to enhance motor neuron survival and delay progress of motor neuron disease. Here, we used NSC‐34 11, a motor neuron‐like cell line, and determined its sensitivity against ferroptosis under normal and differentiated conditions. We showed that NSC‐34 cells became sensitive to ferroptosis during differentiation and identified three factors that are responsible for the enhanced ferroptosis sensitivity in differentiated NSC‐34 cells.

## Materials and methods

### Cell culture

NSC‐34 cells were purchased from Cedarlane (Burlington, NC, USA) (cat. no. CLU140) and maintained in DMEM supplemented with 10% fetal bovine serum (FBS) and penicillin and streptomycin antibiotics (pen/strep). This is the normal growth medium for NSC‐34 cells. For differentiation, cells were harvested using trypsin/EDTA, and cell pellet was washed twice with differentiation medium before seeding into collagen‐coated culture plates (Corning BioCoat, Corning, NY, USA; cat. no. 354400). Four kinds of differentiation media were used in this study: (a) ‘MEM’ – minimum essential medium α (Thermo Fisher Scientific, Waltham, MA, USA; cat. no. 12571063), (b) ‘MEM with atRA’ – MEM with 1 μm all‐*trans*‐retinoic acid (Sigma‐Aldrich, St Louis, MO, USA; cat. no. R2625), (c) ‘F12’ – 1 : 1 of DMEM and Ham's F12 (Corning Cellgro cat. no. 10‐090‐CV), and (d) ‘F12 with atRA’ – F12 medium with 1 μm all‐*trans*‐retinoic acid. All differentiation media were supplemented with 1% FBS, non‐essential amino acids (Thermo Fisher Scientific, cat. no. 11140050), and pen/strep. Cells were incubated in a tissue culture incubator at 37 °C in a humidified incubator containing 5% CO_2_.

### Chemicals and antibodies

Erastin (cat. no. E7781), ferrostatin‐1 (Fer‐1; cat. no. SML0583), propargylglycine (cat. no. P7888), and liproxstatin‐1 (cat. no. SML1414) were purchased from Sigma‐Aldrich. (1*S*,3*R*)‐RSL3 (cat. no. HY‐100218A) and necrostatin‐1 stable (Nec‐1s; cat. no. HY‐14622) were purchased from MedChem Express (Monmouth Junction, NJ, USA), and staurosporine was from LC Laboratories (Woburn, MA, USA) (cat. no. S‐9300). Rabbit antibody against poly(ADP‐ribose) polymerase (PARP) was from Cell Signaling Technology (Danvers, MA, USA; cat. no. 9532), antibody against actin was from Santa Cruz Biotechnology (Dallas, TX, USA; cat. no. sc‐1616‐R), and secondary antibody against rabbit IgG was from LI‐COR Biotechnology (Lincoln, NE, USA; cat. no. 925‐68071).

### Inducing ferroptosis in NSC‐34 cells

Assay plates (Corning, cat. no. 3712) were prepared by seeding 2000 NSC‐34 cells per well in 40 μL of growth medium into black, clear bottom 384‐well plates. The assay plate was incubated for 2 days before compound treatment. On the day of the experiment, empty 384‐well polypropylene plates (Greiner, Monroe, NC, USA; cat. no. 781281) were filled with 50 μL growth medium except for columns 5 where 100 μL of lethal solution (20 μm of erastin and/or RSL3) was transferred. Then, 2‐fold serial dilution of the lethal solution across columns 5–20 was carried out by transferring 50 μL of compound solution to the next column successively with mixing. We named this plate as ‘1× lethal plate’. Culture media in the assay plate were removed, and NSC‐34 cells were treated with lethals in a 2‐fold dilution series by transferring 40 μL solution from a ‘1× lethal plate’. The assay plate was returned to the culture incubator and maintained for 24 h before starting a resazurin viability assay.

### Resazurin viability assay

After 1 day of compound treatment, resazurin (Sigma‐Aldrich, cat. no. R7017) was added to the assay plate to a final concentration of 0.01%. The assay plate was incubated further for 1 day to allow reduction of resazurin, which produced red fluorescence. The fluorescence intensity was determined using a Victor 2 plate reader (Perkin Elmer, Melville, NY, USA) with a 544‐nm excitation filter and a 590‐nm emission filter.

Percentage growth inhibition (% GI) was calculated from the following formula using fluorescence intensity values:

%GI=100∗(1−(X−N)/(P−N))

where *X* is cells treated with compound, *N* is growth medium only, and *P* is cells without any compound.

### Light microscopy

Phase contrast images were obtained using a phase contrast inverted microscope (Motic, Viking Way Richmon, BC, Canada) equipped with a ×10 objective. At least three independent fields were acquired for each experimental condition. Representative photographs from one field of view are shown.

### Analysis of lipid reactive oxygen species generation

NSC‐34 cells were seeded in six‐well plates and treated with test compounds for the indicated time. On the day of experiment, BODIPY™581/591 C11 (Thermo Fisher Scientific; cat. no. D3861) was added to each well to the final concentration of 1.5 μm and the culture plate was incubated for 20 min at 37 °C. Cells were harvested and washed once with Hanks’ balanced salt solution (HBSS; Thermo Fisher Scientific, cat. no. 14025092) to remove excess BODIPY‐C11 dye. After washing, cells were pelleted by spinning, and the cell pellet was resuspended in 500 μL of HBSS. The cell suspension was strained through a 40‐μm cell strainer (BD, San Jose, CA, USA), followed by flow cytometry analysis using Guava^®^ easyCyte Plus (Millipore, Billerica, MA, USA). BODIPY‐C11 signal, which reflects the lipid peroxide level, was measured using the FL1 channel. Experiments were performed in biological triplicates, and a representative result is shown.

### Gene expression analysis by RT‐qPCR

Cells were harvested and washed once with HBSS before freeze storing at −80 °C. On the day of experiment, RNA was purified from the cell pellet using the QIAshredder and RNAeasy extraction kits (Qiagen, Germantown, MD, USA) according to the manufacturer's instructions. Two milligrams of total RNA per sample was subsequently used in a reverse transcription reaction using the TaqMan RT Kit priming with Random Hexamers (Thermo Fisher Scientific). The following TaqMan assay primers were purchased from Thermo Fisher Scientific: *Actb* (assay ID Mm02619580_g1), *Ngfr* (assay ID Mm00446296_m1), *Chat* (assay ID Mm01221880_m1), *Gria1* (assay ID Mm00433753_m1), *Gria2* (assay ID Mm00442822_m1), *Gria3* (assay ID Mm00497506_m1), *Gria4* (assay ID Mm00444754_m1), *Grin1* (assay ID Mm00433790_m1), *Grin2a* (assay ID Mm00433802_m1), *Grin2b* (assay ID Mm00433820_m1), *Grin2d* (assay ID Mm00433822_m1), *Mnx1* (a.k.a. *Hb9*) (assay ID Mm01222622_m1), *Isl1* (assay ID Mm00517585_m1), *Nefm* (assay ID Mm00456200_m1), *Map3k5* (assay ID Mm00434883_m1), *Lamb2* (assay ID Mm00493080_m1), *Itga2* (assay ID Mm00434371_m1), *Col16a1* (assay ID Mm01180622_m1), *Abcc3* (assay ID Mm00551550_m1), *Abca1* (assay ID Mm00442646_m1), *Cth* (assay ID Mm00461247_m1), *Cbs* (assay ID Mm00460654_m1), *Cyp24a1* (assay ID Mm00487244_m1), and *Rarb* (assay ID Mm01319677_m1). Quantitative PCR was performed on triplicate samples in 96‐well format on a Bio‐Rad CFX96 Real‐Time PCR System (Bio‐Rad Laboratories, Hercules, CA, USA). The change in expression of a gene between experimental and control conditions was computed using the ΔΔ*C*_t_ method with *Actb* as an internal reference gene.

## Results

### Induction of ferroptosis in NSC‐34 cells

**Figure 1:**
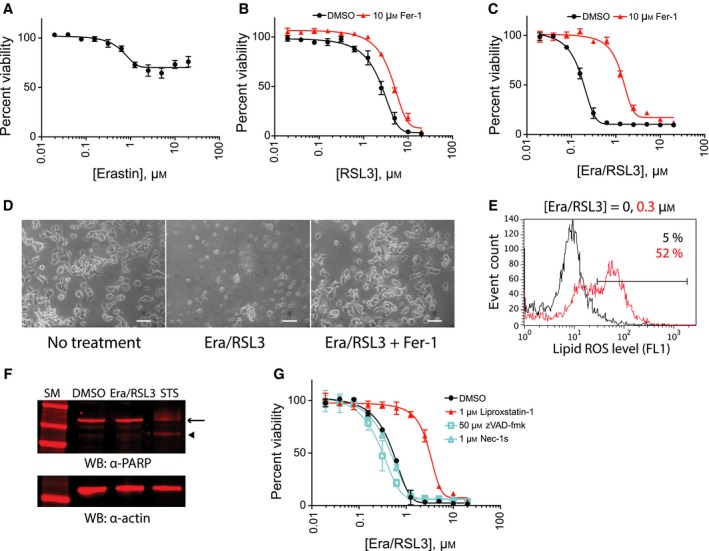
NSC‐34 cells die by ferroptosis upon combination treatment. (A) Erastin treatment inhibited NSC‐34 cell growth but did not kill the cells. (B) RSL3 treatment killed NSC‐34 cells; however, the cell death was not suppressed by ferrostatin‐1 (Fer‐1), a ferroptosis‐specific inhibitor. (C) Combination treatment of erastin (Era) and RSL3 in the presence of 50 μg·mL ^−1^ ferric citrate induced ferroptosis. NSC‐34 cells were treated with indicated amount of ferroptosis inducers for 24 h. Cell viability in (A–C) was determined by fluorimetry with resazurin dye. Data are presented as mean ± SD; *n* = 3. (D) Representative images of NSC‐34 cells that were treated with 0.6 μm of erastin/RSL3 in the presence (right) or absence (middle) of 10 μm Fer‐1. An image of control NSC‐34 cells is also shown (left). Scale bar = 100 μm. (E) Cell death induced by erastin/RSL3 combination treatment accompanied massive generation of lipid peroxides as assessed by BODIPY‐C11 staining and flow cytometry analysis. (F) Western blot with PARP antibody confirmed that cell death by erastin/RSL3 treatment did not accompany PARP cleavage, which is a marker for caspase activation and apoptosis. In contrast, staurosporine, a control apoptosis‐inducing reagent, generated a specific PARP fragment. Arrow indicates full length PARP protein, and arrowhead indicates caspase‐cleaved PARP fragment. The sample western blot membrane was probed with actin antibody to show the loading amount (bottom). Cells were treated with 0.2 μm erastin/RSL3 or 0.01 μm staurosporine. (G) Cell death by erastin/RSL3 was not suppressed by caspase inhibitor (zVAD‐fmk) nor by necroptosis inhibitor (Nec‐1s), but was suppressed by liproxstatin‐1, another ferroptosis inhibitor. NSC‐34 cells were treated with indicated compounds, and cell viability was determined as in (C).

As the sensitivity of NSC‐34 cells against ferroptosis has not been determined yet, we began by testing erastin 3 and RSL3 5, the two canonical ferroptosis inducers, in NSC‐34 cells. Erastin treatment modestly inhibited NSC‐34 cell growth at micromolar range concentration; however, there was no clear cell death, which suggests that NSC‐34 cells are resistant to ferroptosis induced by xCT inhibition (Fig. 1A). The same erastin compound potently killed HT‐1080, a control cell line, as before, confirming the integrity of the erastin batch used in this study 3 (Fig. S1A). NSC‐34 cells expressed the system $x_{c}^{-}$ gene, the target of erastin, as assessed by RT‐qPCR (real time‐quantitative PCR), which excludes the possibility that erastin resistance in NSC‐34 was caused by lack of system $x_{c}^{-}$expression (Fig. S1B).

When RSL3 was supplied, it killed NSC‐34 cells at micromolar range concentration (Fig. 1B). Interestingly, the cell death induced by RSL3 could not be suppressed by ferrostatin‐1 (Fer‐1) (Fig. 1B), which was in sharp contrast to RSL3's activity in HT‐1080 cells where cell death was significantly suppressed by Fer‐1 (Fig. S1C). The expression of GPx4, the target of RSL3, in NSC‐34 cells was confirmed by a RT‐qPCR experiment (Fig. S1B).

It is possible that molecular components required for inducing ferroptosis upon inhibition of xCT or GPx4 are missing or under suboptimal conditions in NSC‐34 cells. We speculated that cellular iron could be such a molecular component because iron plays a critical role in the generation of lipid peroxide either through fenton chemistry 12 or as a cofactor for lipid oxidizing enzymes such as lipoxygenases 13 in a ferroptosis context. Depletion of cellular iron using iron chelators completely suppresses ferroptosis 14. To test this hypothesis, we treated the cells with erastin and RSL3 together in the presence of ferric citrate (Fig. 1C). This combination killed NSC‐34 cells potently, and the cell death was suppressed by Fer‐1 treatment indicating that ferroptosis contributed significantly to this cell death process (Fig. 1C,D). Hereafter, we used an erastin, RSL3, and ferric citrate combination to study ferroptosis in NSC‐34 cells.

We observed a significant increase in lipid peroxidation upon erastin/RSL3 treatment in NSC‐34 cells, which is a hallmark of ferroptosis (Fig. 1E). The cell death produced by erastin/RSL3 did not accompany cleavage of PARP, a representative substrate of cellular caspases, confirming the non‐apoptotic nature of ferroptosis in NSC‐34 cells (Fig. 1F). In addition, the cell death by erastin/RSL3 was not suppressed by a caspase inhibitor [50 μM of benzyloxycarbonyl‐phenylalanyl‐alanyl‐fluoromethyl ketone (zVAD‐fmk)] nor by a necroptosis inhibitor (1 μm of Nec‐1s) whereas liproxstatin‐1, another ferroptosis‐specific inhibitor, was effective in suppressing cell death (Fig. 1G). These data demonstrate that ferroptosis can be induced in NSC‐34 cells by combination treatment of erastin, RSL3, and ferric citrate.

### Determining culture condition that converts NSC‐34 cells into more motor neuron‐like cells

NSC‐34 is a hybrid cell line that was created by fusion of a mouse neuroblastoma cell line (N18TG2) and motor neuron‐enriched spinal cord cells prepared at mouse embryonic day 12–14 11. It was reported that the parental NSC‐34 cells displayed some motor neuron properties, such as generation of action potentials and synthesis of acetylcholine, and can be further differentiated into more motor neuron‐like cells by culturing in ‘differentiation medium’. The first report on NSC‐34 differentiation used a 1 : 1 mixture of DMEM/Ham's F‐12 containing low serum (1% compared to 10% in growth medium) 15. Subsequently, other groups reported successful use of different differentiation medium compositions 16, 17. They had low serum in common but either had retinoic acid additive or used different basal medium such as MEM or DMEM. Since differentiation behavior of NSC‐34 cells may vary according to the passage number or source of origin, we monitored changes in the cell morphology and the expression of motor neuron markers upon culturing NSC‐34 cells in four distinct differentiation media.

**Figure 2:**
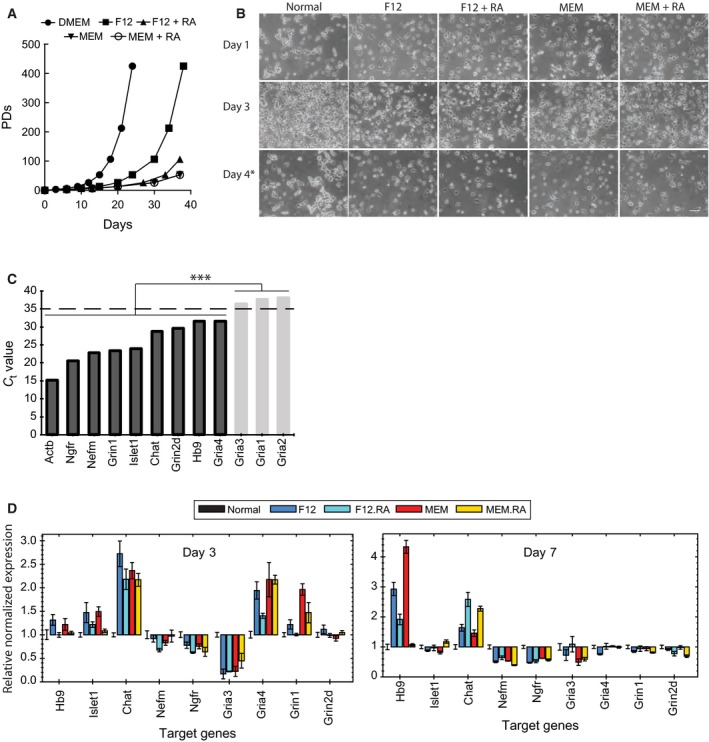
NSC‐34 cells became motor neuron‐like cells by culturing in differentiation media. (A) NSC‐34 cells in differentiation media grew slower than in normal culture medium. PDs, population doublings. (B) Representative images of NSC‐34 cells in normal and differentiation media. Compared to cells in normal medium, cells in differentiation media have elongated structure resembling neuronal processes. Images were taken at 1, 3, and 4 days after growing NSC‐34 cells in respective differentiation media. Scale bar = 100 μm. (C) RT‐qPCR analysis of motor neuron marker genes in NSC‐34 cells grown in normal medium. Genes whose expression level was too low (*Gria3*,* Gria1*, and *Gria2*) were excluded from further analysis. (D) Changes in motor neuron marker gene expression level upon NSC‐34 differentiation. RNA samples were prepared from NSC‐34 cells grown in either normal medium (Normal) or differentiation media (F12, F12 + RA, MEM, or MEM + RA), and gene expression level was determined by RT‐qPCR. Gene expression level in differentiation condition was normalized to that in normal condition and is presented as fold change. ***P < 0.001. Data in (D) are presented as mean ± SD; *n* = 3.

NSC‐34 cells in normal growth medium (10% FBS in DMEM) grew fast requiring two passages per week on average (Fig. 2A). On the other hand, NSC‐34 cells in differentiation media showed slower growth rate and contained cells with neuron‐like projections (Fig. 2A,B). The morphological distinction between parental NSC‐34 and differentiated NSC‐34 cells was the most evident when the culture was passaged to avoid over‐confluence (Fig. 2B, day 4). We note that we had to split differentiated culture once in 7–10 days, which was different from the reported property of NSC‐34_D_ that did not require subculture passage once differentiation starts 15, 17.

We wanted to find the best differentiation condition by morphological analysis such as length of neuron‐like projections; however, NSC‐34 cells looked very similar in all four conditions (Fig. 2B). Therefore, we decided to perform a RT‐qPCR analysis to examine expression of motor neuron markers. These included two motor neuron lineage markers (Hb‐9 and Islet‐1), one cholinergic marker (Chat), one growth factor receptor (p75NTR), one neurofilament marker (neurofilament‐M), and eight glutamate receptors (Gria1–4, Grin1, Grin2a, Grin2b, and Grin2d). Total RNAs were harvested at day 3 and day 7 after differentiation, and expression level of each motor neuron marker gene was examined (Fig. 2C).

Parental NSC‐34 cells strongly expressed four neuronal markers: neurotrophin receptor (*Ngfr*), neurofilament medium chain (*Nefm*), glutamate ionotropic receptor NMDA type subunit 1 (*Grin1*), and ISL LIM homeobox 1 (*Islet1*) (Fig. 2C). In contrast, there was very poor or no expression of five glutamate receptor genes – *Gria1*,* Gria2*,* Gria3*,* Grin2a*, and *Grin2b* – in the parental NSC‐34 cells (Fig. 2C; note that the *C*_t_ value of *Grin2a* and *Grin2b* could not be determined due to the lack of expression). We could not detect significant expression of these five glutamate receptor genes throughout various time points and differentiation conditions; therefore, we excluded these genes from further analysis. Four other neuronal markers (*Chat*,* Grin2d*,* Hb9*,* Gria4*) showed intermediated expression level in NSC‐34 cells and were included in our next analysis.

For the eight marker genes with good expression level, we determined fold‐changes in the level of expression between differentiation and normal culture conditions. The motor neuron marker genes displayed varying levels of expression depending on the composition of differentiation media and the timing of differentiation (Fig. 2D). Consistent upregulation of the choline *O*‐acetyltransferase gene (*Chat*) was notable indicating that the gene was expressed in all four differentiation conditions. *Hb9*, a motor neuron lineage gene, was slightly upregulated on day 3, and then further upregulated on day 7. It is likely that the upregulation of the *Hb9* gene drives motor neuron‐like phenotypes of NSC‐34 under differentiation conditions. *Islet1*, the other motor neuron lineage gene, was also slightly upregulated on day 3 but the upregulation did not last for a longer time. Because the basal expression level of *Islet1* was high in NSC‐34 cells (Fig. 2C), it is possible that a further increase in *Islet1* expression could not occur. On the other hand, it is puzzling to see downregulation of the neurofilament medium gene (*Nefm*) and the neurotrophin receptor gene (*Ngfr*;* p75NTR*) under differentiation conditions. Also, individual glutamate receptor genes responded differently upon each differentiation condition. The results indicate that the differentiation conditions could drive NSC‐34 cells to display some aspects of motor neurons but not all features. We selected MEM medium and day 3 time point to compare ferroptosis sensitivity between normal and differentiation conditions because motor neuron markers were more upregulated in that condition (Fig. 2D).

### Differentiation of NSC‐34 cells made cells more sensitive to ferroptosis

NSC‐34 cells were differentiated by culturing cells in MEM with 1% serum medium for 3 days, and then the differentiated NSC‐34 cells were exposed to erastin/RSL3 in a 2‐fold dilution series in the presence of ferric citrate to induce ferroptosis. As a control, NSC‐34 cells were cultured in normal medium with ferric citrate and treated with erastin/RSL3 in parallel to compare ferroptosis sensitivity.

**Figure 3:**
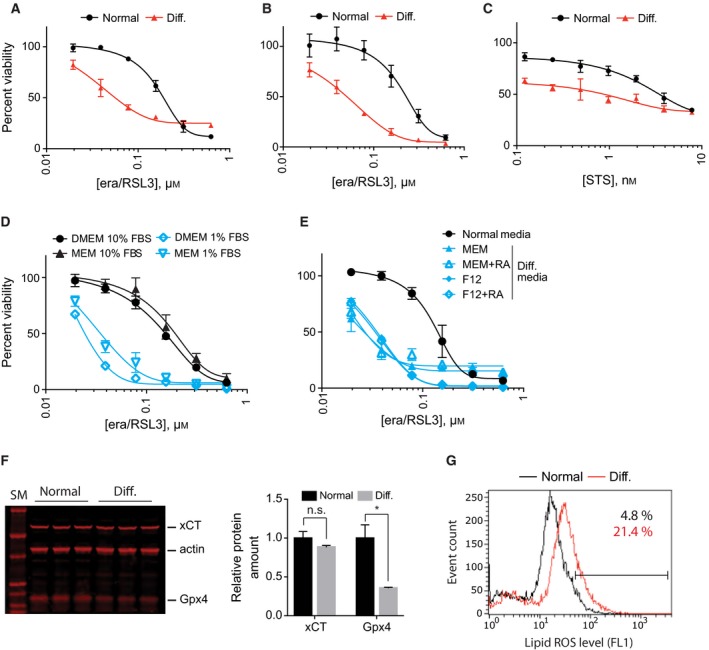
NSC‐34 cells became more sensitive to ferroptosis upon differentiation. (A) Comparison of ferroptosis sensitivity between NSC‐34 cells grown in normal and differentiation media. Cell viability was determined by fluorimetry using resazurin dye. (B) Same as in (A) except that cell viability was determined by luminometry using RealTime‐Glo reagent. (C) Upon differentiation, NSC‐34 cells became sensitive to staurosporine treatment as well, which indicates that sensitization effect is not specific to ferroptotic cell death. (D) Low serum (1%) in differentiation media caused ferroptosis sensitization in NSC‐34 cells. (E) NSC‐34 cells became sensitive to ferroptosis in all four differentiation conditions because they all contain low serum. NSC‐34 cells were treated with indicated amount of lethal compounds for 24 h. Cell viability in (A–E) was determined using resazurin viability dye. Data are presented as mean ± SD; *n* = 3. (F) Western blot analysis showed GPx4 expression was decreased in differentiation condition while the xCT level did not change. Quantifications of the results are shown in the graph. Data are presented as mean ± SD; *n* = 3. n.s., not significant; *P < 0.05 by Welch's *t* test. (G) NSC‐34 cells showed higher level of lipid peroxides in differentiation condition. BODIPY‐C11 dye was used to determine lipid peroxide level.

We observed that differentiated NSC‐34 cells became more sensitive to erastin/RSL3 treatment compared to undifferentiated NSC‐34 cells (Fig. 3A). The sensitization effect was not an artifact from the resazurin assay because viability determination using an independent reagent, RealTime‐Glo™ (Promega Corp., Madison, WI, USA), showed the same sensitization effect (Fig. 3B). However, this sensitization effect was not specific to erastin/RSL3. Differentiated NSC‐34 cells became more sensitive to staurosporine, a control apoptosis‐inducing reagent as well (Fig. 3C). The results suggest that cellular changes in NSC‐34 upon differentiation were very broad and caused sensitization to diverse lethal stimuli.

The composition of differentiation medium was MEM with 1% fetal bovine serum (FBS) whereas that of normal medium was DMEM with 10% FBS. It is likely that low serum in the differentiation medium made cells sensitive to ferroptosis because serum contains plenty of nutrients and antioxidants. To test this hypothesis, we cultured NSC‐34 cells in MEM with 1% FBS, DMEM with 1% FBS, or MEM with 10% FBS and compared sensitivity against erastin/RSL3. As expected, erastin/RSL3 was more potent in medium with 1% FBS regardless of composition of basal medium (DMEM or MEM) (Fig. 3D). We extended our analysis using the four media that can induce motor neuron differentiation in NSC‐34 cells (Fig. 2). Since low serum (1% FBS) was used in all four conditions, it was expected that NSC‐34 cells would become sensitive to ferroptosis in all four conditions, which turned out to be the case (Fig. 3E). It is notable that retinoic acid, a well‐known neurogenic factor 18, did not make any difference in ferroptosis sensitivity in our experiment. The effect of retinoic acid in NSC‐34 differentiation was negligible too (Fig. 2). We speculate that the effect of retinoic acid on ferroptosis, if any, may be revealed in a model other than NSC‐34 where the neurogenic effect of retinoic acid is more evident.

One mechanism that made NSC‐34 cells sensitive to ferroptosis upon low serum condition could be downregulation of xCT or GPx4 because these two proteins are the targets for erastin and RSL3, respectively. Interestingly, western blot analysis showed that there was less GPx4 protein in the differentiation condition compared to the normal condition (65% reduction relative to normal condition, Fig. 3F). This downregulation took place at the post‐transcriptional level since we could not detect any differences in *GPx4* mRNA level between normal and differentiation conditions (see RNA‐seq data below). In contrast, there was no significant change in the amount of xCT (Fig. 3F).

It is likely that insufficient amount of serum antioxidants and reduction in GPx4 enzyme could lead to elevation of basal oxidative stress. In an engineered fibroblast cell line overexpressing HRAS^G12V^, basal reactive oxygen species (ROS) level was elevated, which accounted for enhanced sensitivity against ferroptosis 5, 19. To test this, we compared basal lipid‐ROS level in NSC‐34 cells that were grown in normal medium (DMEM with 10% FBS) and in differentiation medium (MEM with 1% FBS). NSC‐34 cells in the differentiation condition contained a higher level of lipid‐ROS compared to NSC‐34 cells in normal medium (21.4% *vs* 4.8% in BODIPY‐C11 positive population), which supports our hypothesis that elevated oxidative stress under the differentiation condition made NSC‐34 cells more susceptible to ferroptosis (Fig. 3G).

### Involvement of transsulfuration pathway in NSC‐34 cell ferroptosis

While low serum antioxidants contributed as a cell extrinsic factor in ferroptosis sensitization, it is also possible that culturing NSC‐34 cells under a low serum condition induced cell intrinsic changes that also contributed to the ferroptosis sensitivity such as reduction in GPx4 protein amount. In order to identify those cell‐intrinsic changes, we prepared mRNA samples from NSC‐34 cells in normal and differentiation media and carried out an RNA‐Seq experiment to determine changes in individual gene expression level during the differentiation process. For the differentiation sample, NSC‐34 cells were cultured in MEM‐based differentiation medium for 3 days followed by mRNA extraction.

**Figure 4:**
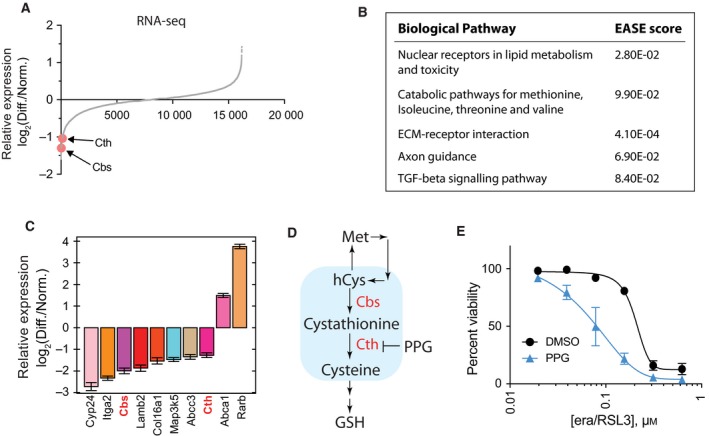
Inhibition of transsulfuration pathway contributed to ferroptosis sensitization. (A) Global changes in gene expression level during NSC‐34 cell differentiation were determined by RNA‐seq analysis. (B) Gene set enrichment analysis of the RNA‐seq data in (A) revealed five biological pathways affected by the differentiation process in NSC‐34. (C) The expression changes in the selected genes were confirmed by RT‐qPCR analysis. These genes were selected from gene set enrichment analysis in (B). (D) A simplified diagram of transsulfuration pathway. Two genes, *Cbs* and *Cth*, that were detected in RNA‐seq analysis are colored red. Propargylglycine (PPG), a known transsulfuration pathway inhibitor, is also shown. hCys, homocysteine; GSH, reduced glutathione. (E) Inhibition of transsulfuration pathway using PPG made NSC‐34 cells sensitive to ferroptosis. NSC‐34 cells in normal medium were treated with erastin/RSL3 in the presence or absence of PPG for 24 h. Cell viability was determined by fluorimetry with resazurin dye. Data are presented as mean ± SD; *n* = 3.

Our RNA‐Seq experiment was able to analyze expression of 19 159 genes in total (Fig. 4A and Table S1). Interestingly, we observed only small changes in the overall gene expression level during the NSC‐34 differentiation process. For example, the *Oprd1* gene was upregulated by 2.16‐fold during the differentiation and that was the most upregulated gene during NSC‐34 differentiation. Similarly, the *Rprml* gene was downregulated by 2.52‐fold and that was the most downregulated gene in our analysis. Therefore, we focused on finding biological pathways affected during the differentiation process, and required more than 2‐fold changes in the gene expression level between the two conditions, which resulted in selecting 42 upregulated and 145 downregulated genes (see Table S1 for the list of selected genes).

We uploaded the list of selected genes to the DAVID bioinformatics analysis platform 20 in order to identify biological pathways enriched in the gene set. DAVID returned several biological pathways that passed cutoff value of EASE score (a modified Fisher's exact test) 20 when default analysis parameters were used. These included ‘nuclear receptors in lipid metabolism and toxicity’ and ‘catabolic pathways for methionine, isoleucine, threonine, and valine’ (Fig. 4B). We performed a RT‐qPCR experiment and confirmed that differential expression of genes associated with these two biological pathways were reproducible (Fig. 4C).

We were particularly interested in the ‘catabolic pathways for methionine, isoleucine, threonine, and valine’ category because DAVID analysis returned this pathway based on significant downregulation of two genes, *Cbs* and *Cth*, upon NSC‐34 cell differentiation (Fig. 4A)*. Cbs* and *Cth* encode cystathionine‐β‐synthase and cystathionine‐γ‐lyase, respectively, which catalyze the first two committed steps in the transsulfuration pathway (Fig. 4D) 21. The transsulfuration pathway is a metabolic pathway that involves interconversion of cysteine and homocysteine, and is known to regulate ferroptosis 22. Since both the *Cbs* and *Cth* genes were downregulated, it is likely that the transsulfuration pathway became inhibited during NSC‐34 differentiation. In order to test whether inhibition of the transsulfuration pathway contributed to ferroptosis sensitization during NSC‐34 differentiation, we used propargylglycine (PPG), a well‐known inhibitor of the transsulfuration pathway, and monitored ferroptosis sensitivity. Indeed, PPG treatment made NSC‐34 cells more sensitive to ferroptosis induced by erastin/RSL3 (Fig. 4E). The results support our hypothesis that inhibition of the transsulfuration pathway during NSC‐34 differentiation contributed to the ferroptosis sensitization.

## Discussion

Using the NSC‐34 cell line, we tested whether the ferroptosis pathway is operating under a motor neuron background and found that ferroptosis could be induced in NSC‐34 cells by combination treatment of erastin, RSL3, and ferric citrate (Fig. 1). Due to the oxidative nature of ferroptosis, it is often unclear whether ferroptosis is similar to necrotic cell death induced by a high dose of oxidizing biocides such as hydrogen peroxide. Hydrogen peroxide‐induced necrosis damages numerous targets within a cell and cannot be suppressed by ferroptosis‐specific inhibitors such as Fer‐1 3. In contrast, ferroptotic cell death can be suppressed by lipophilic antioxidants (Fer‐1 and vitamin E) and can be regulated by a distinctive set of genes 3, 23. In this context, induction of ferroptosis in NSC‐34 cells upon a combination treatment, where an individual reagent was not effective, reflects the regulated nature of ferroptotic cell death.

NSC‐34 cells became more sensitive to ferroptosis in differentiation conditions (Fig. 3). We identified that low serum (one‐tenth of normal growth medium) in differentiation media was the main driving factor for the sensitization effect (Fig. 3). Furthermore, the RNA‐seq experiment suggested that inhibition of the transsulfuration pathway contributed to ferroptosis sensitization under differentiation conditions (Fig. 4). It is unclear how differentiation conditions downregulated *Cbs* and *Cth*, the two enzymes catalyzing committed steps of transsulfuration pathway in NSC‐34 cells. Similar to our results, *Cth* expression was downregulated, and the transsulfuration pathway was inhibited in a model of kidney ischemia–reperfusion injury 24. This is interesting because the functional role of ferroptosis in kidney injury has been demonstrated in several models 25, 26, 27. Modulation of the transsulfuration pathway may be exploited to provide a protective effect during kidney injury. For example, activation of the transsulfuration pathway played a protective role by upregulating the cellular glutathione level in several models 28, 29.

Our data as well as data from GPx4 knockout mouse models raise an interesting question – why are neurons in general so sensitive to ferroptosis? Neuronal membrane lipids are enriched with polyunsaturated fatty acid (PUFA) side chains, especially those of arachidonic acids and docosahexaenoic acids 30. PUFAs are vulnerable to iron‐mediated lipid peroxide generation and those lipid peroxides drive ferroptotic cell death. Addition of PUFAs to the culture medium made HT‐1080 cells more sensitive to ferroptosis, whereas addition of deuterated PUFAs that prevent lipid peroxide generation suppressed ferroptosis 31. Therefore, enriched PUFAs is one factor that can explain ferroptosis sensitivity in neurons. The second sensitivity factor in neurons could be iron. Higher iron content has been detected in several brain areas such as substantia nigra, caudate nucleus, and putamen 32. Enzymes catalyzing the first steps in the synthesis of neurotransmitters such as dopamine and serotonin use iron as a critical cofactor, which may explain why neurons have a higher level of iron. Depletion of cellular iron using iron chelator significantly suppressed ferroptosis 14, whereas addition of iron made cells more sensitive to ferroptosis 3. The contribution of iron in neuronal ferroptosis should be more evident in neuropathological conditions since damaged neurons release iron ions in forms capable of catalyzing lipid peroxidation. The third sensitivity factor in neurons may be excitotoxic amino acids (EAAs) such as glutamate. EAAs are released from many neurons and account for most excitatory synaptic activity in the central nervous system. Binding of EAAs to respective ionotropic receptors results in prolonged increase in Ca^2+^ and reactive oxygen species and nitrogen species. Therefore, neurons have a higher level of oxidative stress during normal activity, which may account for the higher sensitivity to ferroptosis in general.

The NSC‐34 cell line has been used in studying mechanisms for motor neuron diseases such as amyotrophic lateral sclerosis 33, 34, 35. Compared to other *in vitro* motor neuron models, NSC‐34 is much easier to grow making it a cell line of choice for initial testing. However, the usefulness of this model has been repeatedly questioned because the cell line displays only partial characteristics of motor neurons. As pointed out by Madji Hounoum *et al*. 17, we also found that the phenotype of NSC‐34 cells changes upon prolonged and repeated culture passages. We were able to resolve the issue by using fresh frozen stock and not maintaining the NSC‐34 culture for more than 1 month. These and other limitations of the NSC‐34 model should be considered carefully in determining the suitability of this model for testing any hypothesis.

## Conflict of interest

The authors declare no conflict of interest.

## Author contributions

Conceived and designed the experiments: WSY. Performed the experiments and analyzed the data: AMM, JM, ZS, FP, and WSY. Wrote the paper: AMM and WSY.

## Supporting information


**Fig. S1.** (A) Erasin killed HT‐1080, a ferroptosis control cell line, as before indicating that erastin batch we are using is good. (B) RT‐qPCR analysis was used to confirm expression of *GPx4* and *xCT*, the target of RSL3 and erastin, in NSC‐34 cells. The figure shows the amplification plot of each gene. Triplicate samples were analyzed for each gene using mRNA preparation from NSC‐34 cells. The black lines represent amplification of Actb, a housekeeping gene. (C) Cell death induced by RSL3 was significantly suppressed by Fer‐1, a ferroptosis specific inhibitor. HT‐1080 cells were treated with indicated amount of ferroptosis inducers for 24 h. Cell viability in was determined by fluorimetry with resazurin dye. Data were presented as mean ± SD; *n* = 3.

**Table S1.** RNASeq analysis of differentially expressed genes during NSC‐34 differentiation. Yellow color highlights 145 genes whose expression was downregulated during NSC‐34 differentiation by more than 2‐fold. Blue color highlights 42 genes whose expression was upregulated during NSC‐34 differentiation by more than 2‐fold. These 187 genes were used for pathway analysis in DAVID.
