# Supplementary material for: NSC‐34 motor neuron‐like cells are sensitized to ferroptosis upon differentiation
Source: FEBS Open Bio. 2019 Feb 23;9(4):582–93. doi: 10.1002/2211-5463.12577 (PMC6443867; doi:10.1002/2211-5463.12577)
Supplement: Supplementary file 1 — Fig. S1. (A) Erasin killed HT‐1080, a ferroptosis control cell line, as before indicating that erastin batch we are using is good. (B) RT‐qPCR analysis was used to confirm expression of GPx4 and xCT, the target of RSL3 and erastin, in NSC‐34 cells. The figure shows the amplification plot of each gene. Triplicate samples were analyzed for each gene using mRNA preparation from NSC‐34 cells. The black lines represent amplification of Actb, a housekeeping gene. (C) Cell death induced by RSL3 was significantly suppressed by Fer‐1, a ferroptosis specific inhibitor. HT‐1080 cells were treated with indicated amount of ferroptosis inducers for 24 h. Cell viability in was determined by fluorimetry with resazurin dye. Data were presented as mean ± SD; n = 3. [file FEB4-9-582-s001.pdf]

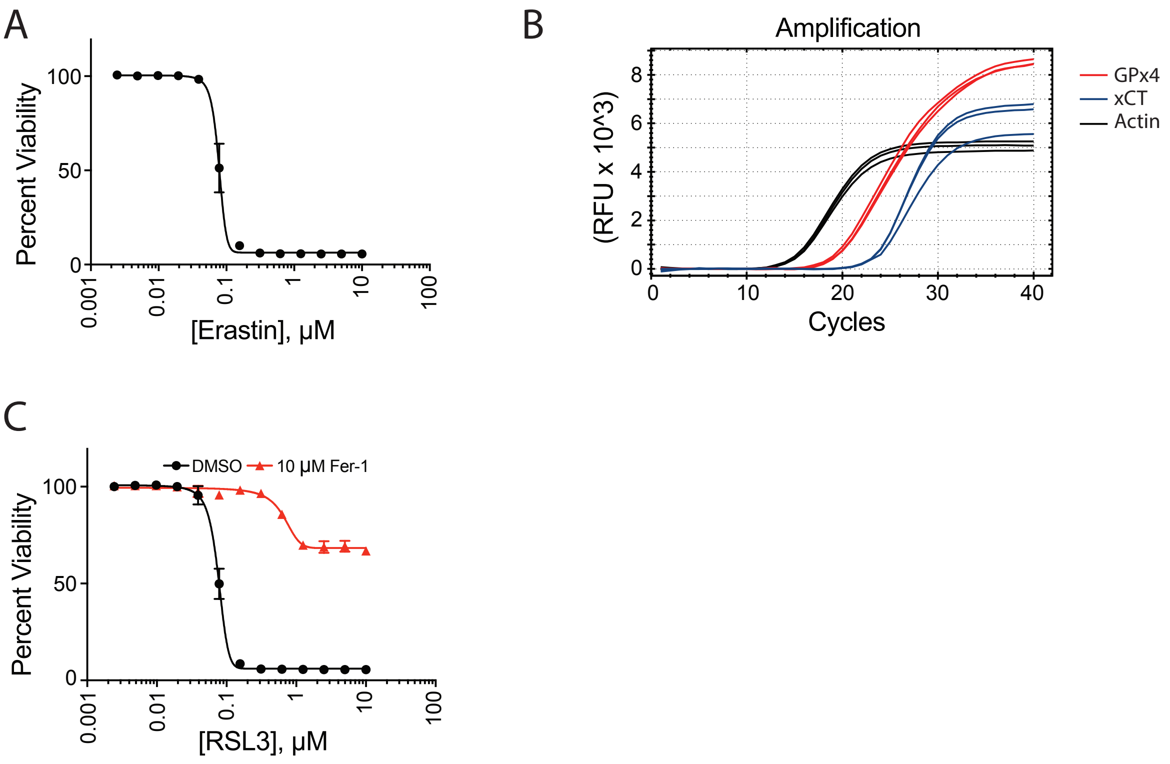

Supplementary Fig. 1. (A) Erasin killed HT-1080, a ferroptosis control cell line, as before indicating that erastin batch we are using is good. (B) RT-qPCR analysis was used to confirm expression of GPx4 and xCT, the target of RSL3 and erastin, in NSC-34 cells. The figure shows the amplification plot of each gene. Triplicate samples were analyzed for each gene using mRNA preparation from NSC-34 cells. The black lines represent amplification of *Actb*, a housekeeping gene. (C) Cell death induced by RSL3 was significantly suppressed by Fer-1, a ferroptosis specific inhibitor. HT-1080 cells were treated with indicated amount of ferroptosis inducers for 24 hrs. Cell viability in was determined by fluorimetry with resazurin dye. Data were presented as mean ± s.d.; n = 3.
